# A Novel Cyclodextrin-Functionalized Hybrid Silicon Wastewater Nano-Adsorbent Material and Its Adsorption Properties

**DOI:** 10.3390/molecules23061485

**Published:** 2018-06-19

**Authors:** Jing Li, Chao Qiu, Haoran Fan, Yuxiang Bai, Zhengyu Jin, Jinpeng Wang

**Affiliations:** 1 State Key Laboratory of Food Science and Technology, Jiangnan University, Wuxi 214122, China; 18246037016@163.com (J.L.); 930017357@163.com (C.Q.); fanhaoran0622@163.com (H.F.); ybai@jiangnan.edu.cn (Y.B.); fpcenter@jiangnan.edu.cn (Z.J.); 2 School of Food Science and Technology, Jiangnan University, Wuxi 214122, China; 3 Synergetic Innovation Center of Food Safety and Nutrition, Jiangnan University, Wuxi 214122, China

**Keywords:** β-cyclodextrin, hybrid silicon, nano-adsorbent material, adsorption

## Abstract

A novel cyclodextrin-functionalized hybrid silicon nano-adsorbent material (6-EA-β-CD-Si) was synthesized via the nucleophilic substitution method. The structure was detected by Fourier transform infrared (FT-IR), X-ray, thermogravimetric analysis, and Brunauer-Emmett-Teller (BET) analysis. Results reveal that the BET surface area of 6-EA-β-CD-Si is 240 m^2^/g and the average pore size is 4.16 nm. The adsorption properties of 6-EA-β-CD-Si onto methylene blue (MB) were studied and fitted with adsorption kinetic models. Both the Freundlich adsorption isotherm model and pseudo-second-order model were fitted with well shows that the multi-layer adsorption with chemisorption and physisorption co-existing in the system. The maximum adsorption capacities are 39.37, 39.21, 36.90, and 36.36 mg/g at temperatures 303, 313, 323, and 333 K, respectively. The maximum removal rate of MB could reach 99.5%, indicating the potential application value of 6-EA-β-CD-Si in wastewater treatment. The adsorption mechanisms of 6-EA-β-CD-Si showed that the hydrophobic cave of β-CD plays an important role on the adsorption of MB.

## 1. Introduction

Water pollution from small industries is a serious problem throughout the world [1]. Toxic dyeing effluents generated in the textile, dyestuffs, leather, papermaking, and plastics are genotoxic, carcinogenic, and teratogenic hazards to humans and pose a significant threat to human health [2]. Therefore, removing the dye in wastewater to an acceptable level before being discharged into the natural environment has been defined in many countries’ laws. To date, a series of methods have been adopted, including catalytic degradation [3], biological treatment [4], chemical precipitation [5], membrane filtration [6], adsorption [7], and many other technologies to reduce the pollution and hazard to the environment caused by dye wastewater. However, each method has its own limitations. At present, the adsorption process is considered as one of the most effective and economical methods for removing pollutants from effluent due to its simplicity of design and ease of operation [8,9]. Some of easy acceptable adsorbent materials such as zeolite [10], graphene [11], metal oxides [12], magnetic particles [13], and activated carbon [14] have been chosen for removing dyes in wastewater. However, there are still some limitations for these materials being used widely because of slow adsorption rate, low adsorption capacity, and inconvenient separation [15]. Therefore, high selectivity, high adsorption capacity, long useful lifetime, and low-cost promising adsorbents are still in urgent need.

It is widely known that β-cyclodextrin (β-CD) is a commercial natural macrocycle molecule that contains a hydrophilic exterior and a hydrophobic internal cavity and has properties of non-toxicity and biodegradability due to its special structure and excellent chemical reactivity. β-CD can form a complex with a wide range of organic and inorganic molecules, charged ions, and other groups in a solution by host-guest interactions through hydrophobic interactions and hydrogen-bonding interactions [16]. However, β-CD was hard to apply in wastewater directly because of its solubility in water. Fortunately, a large number of hydroxyl groups on the surface of β-CD can undergo chemical reactions to improve its performance and to gain insoluble properties [17]. It has been used as a drug carrier [18], to remove metals [19] and organic compounds [20] from aqueous media, as well as in catalysis [21], and chromatography [22]. Therefore, they can also enable relatively facile synthesis of innovative materials for applications in the field of pollution removal from various compartments of the environment such as water, air and soil. However, the preparation steps of the traditionally modified cyclodextrin are quite complicated, with large amounts of reagents used, the cost is relatively high, and the yield of the intermediate product is low [23]. The latest application of nanomaterials in wastewater treatment provided us with the idea to construct a kind of structure in accordance with the nanoscale adsorbent based on the covalent immobilization of β-CD [24]. In this study, a new way was found to solve these problems by making a novel nanoparticle adsorbent.

The novel nanoparticle adsorbent was made by immobilization of β-CD on hybrid silicon by the nucleophilic substitution method, with hybrid silicon inside as the core and β-CD as the “shell.” The most important aspects for this novel material was that the β-CD shell should avoid the violent changes or even destruction of the cavity. Furthermore, the hybrid silicon as carrier and core play the role of immobilization and as an adsorbing material because of its large surface area, large pore volume, and good performance. Both the core and “shell” in this novel nanomaterial are capable of organic pollutants adsorption [25]. It can also overcome the problems of water-solubility, difficulties of recovery, and other defects and achieve a synergism effect between β-CD “shell” and the carrier core [26]. The adsorption ability was evaluated for the treatment of methylene blue (MB) wastewater. This novel material combined the advantages of both hydrophobic cavity of β-CD and high specific surface area of hybrid silicon, and it has the advantage of being environmentally friendly, having low costs in production, strong mechanical stability, and it is easy to separate from the wastewater.

## 2. Results and Discussion

### 2.1. Structural Characterization

**Scheme 1:**
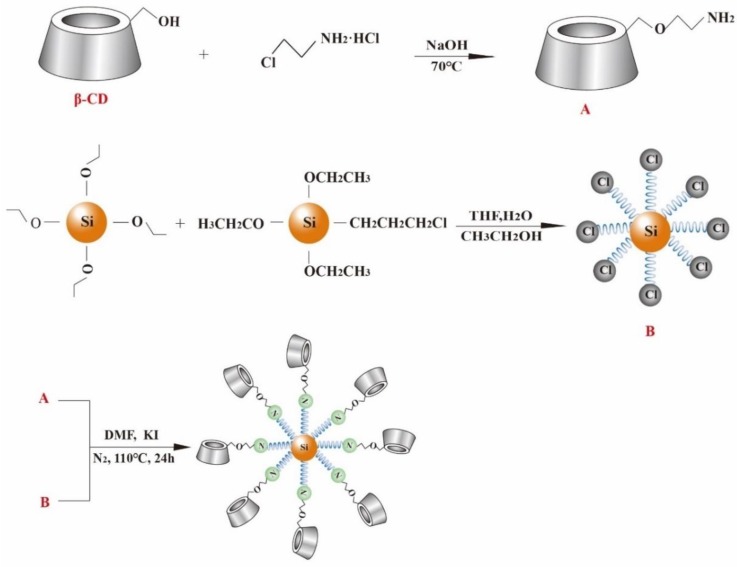
Schematic representation of the preparation steps of 6-EA-β-CD-Si.

**Figure 1:**
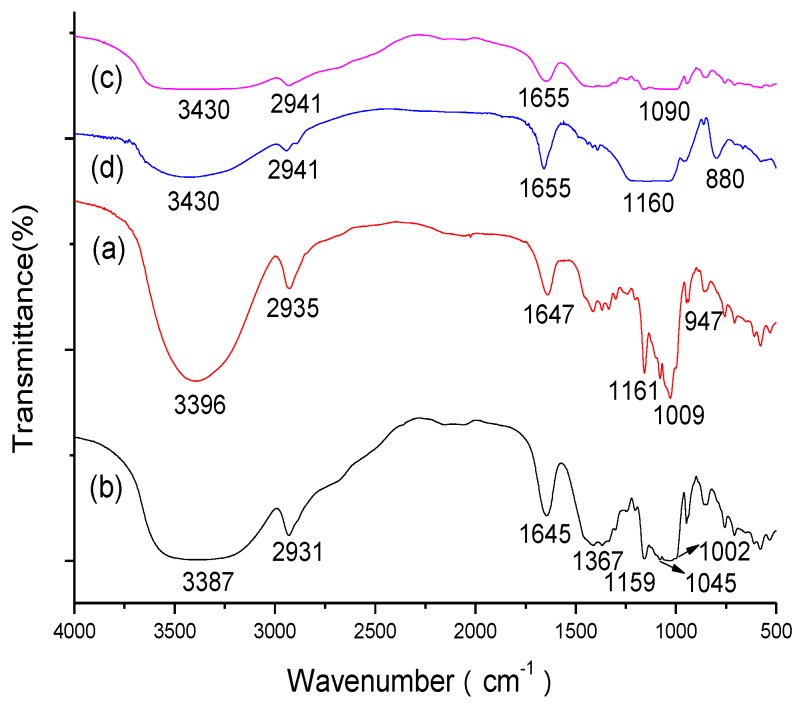
Fourier transform infrared (FT-IR) spectra of β-CD (a), 6-EA-β-CD (b), Cl-Si (c), and 6-EA-β-CD-Si (d).

The overall synthesis process is divided into three steps, and the corresponding synthetic route is shown in Scheme 1. The synthesis of 6-EA-β-CD was achieved through the formation of ether linkage between hydroxyl group and halogen. The 6-EA-β-CD-Si forms secondary amino groups via nucleophilic substitution. However, as a novel particle, the exact structure information was unknown. The linkage, crystallize, structure and geometry shape were detected as follows:

The formation of secondary amine groups is the key step in the synthesis of 6-EA-β-CD-Si. Thus, the Fourier transform infrared (FT-IR) spectra of β-CD, 6-EA-β-CD, Cl-Si and 6-EA-β-CD-Si are illustrated in Figure 1. Comparing the FT-IR spectra of β-CD and 6-EA-β-CD, the strong and broad peaks at around 3396 cm^−1^ are attributed to the O-H stretching vibrations, and the peak becomes wider and broader on the FT-IR spectra of 6-EA-β-CD. The peaks at around 2900 cm^−1^ belong to the C-H stretching vibrations, and the N-H deformation vibration at around 1645 cm^−1^ appeared in 6-EA-β-CD for the existence of the primary amine groups. Meanwhile, the peaks at 1045 cm^−1^ were attributed to the C-O-C stretching vibrations, and the peaks at 1159 cm^−1^ corresponded to the C-C and C-O stretching vibrations [27]. The peak at 1367 cm^−1^ was attributed to the C-N stretching vibration, and red shift occurs on the FT-IR spectra of 6-EA-β-CD due to the amine ethyl was grafted on β-CD, which verified that 6-EA-β-CD molecules were conjugated via the nucleophilic substitution method. Furthermore, comparing with the FT-IR spectroscopy of Cl-Si and 6-EA-β-CD-Si, the O-H stretching vibration in 6-EA-β-CD-Si become stronger due to the abundant hydroxyl group existing of β-CD molecules. The peaks at around 1160 cm^−1^ belong to the C-C and C-O stretching vibrations. The peak at 880 cm^−1^ corresponded to the bending vibration of the R-1,4-bond in β-CD. Meanwhile, the N-H deformation vibration at around 1655 cm^−1^ become stronger in 6-EA-β-CD-Si for the existence of the secondary amine groups. Thus, the FT-IR spectroscopy characterization proves that β-CD is successfully immobilized on the surface of Cl-Si. 

**Figure 2:**
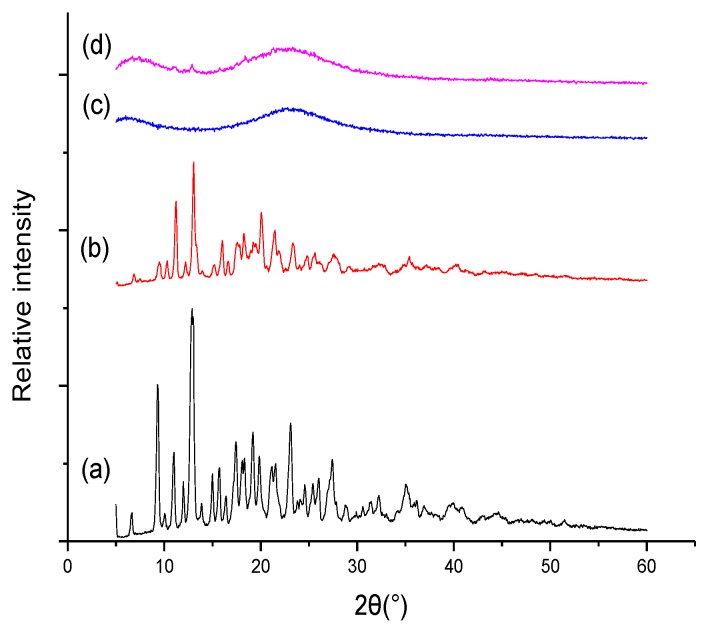
X-ray diffraction (XRD) spectra of β-CD (a), 6-EA-β-CD (b), Cl-Si (c) and 6-EA-β-CD-Si (d).

According to the results of X-ray diffraction (XRD) (Figure 2), it can be derived that there are many characteristic diffraction peaks of β-CD and 6-EA-β-CD in the range of 2*θ* = 10–25°. These peaks are indicative of the presence of highly crystalline [28]. Meanwhile, it was found that the crystallization peak of 6-EA-β-CD slightly lower than that of β-CD, which is due to the substitution of the aminoethyl group of β-CD by the aminoethyl group, and it is similar to the result of grafting citric acid on the surface of β-CD to form cyclodextrin derivatives [29]. Meanwhile, it is noted that Cl-Si and 6-EA-β-CD-Si have a wider peak shape in the range of 2*θ* = 20–25°, which mainly shows the characteristic peak of Cl-Si. After 6-EA-β-CD is immobilized on the surface of Cl-Si, the overall peaks of Cl-Si had almost not changed, mainly because β-CD is arranged in the monomolecular layer on the surface of Cl-Si and there are not enough molecules to form β-CD crystal structures, so it is difficult to observe the β-CD characteristic peaks from the XRD of 6-EA-β-CD-Si. The X-ray diffraction characterization further proves that β-CD is successfully immobilized on the surface of Cl-Si.

**Figure 3:**
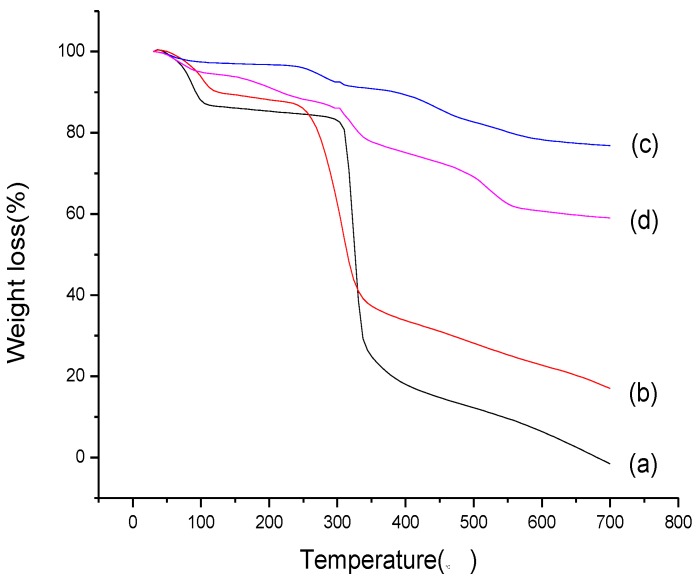
TG curves of β-CD (a), 6-EA-β-CD (b), Cl-Si (c) and 6-EA-β-CD-Si (d).

According to the TG curves (Figure 3), the weight loss before 150 °C comes from the moisture on the sample surface and inside the cavity of β-CD. Comparing β-CD with 6-EA-β-CD, the peak temperature of 6-EA-β-CD and the temperature corresponding to 50% weight loss of 6-EA-β-CD were lower than that of β-CD, which is mainly due to the higher disorder of the cyclodextrin molecules after grafting of the amine ethyl group on β-CD [19]. Meanwhile, the reduction of the crystallinity of 6-EA-β-CD in the X-ray spectrum also confirms this phenomenon. Compared to Cl-Si and 6-EA-β-CD-Si, the weight loss of Cl-Si was only 3% before 150 °C, which is mainly due to the hydrophobic surface of Cl-Si, while the weight loss of 6-EA-β-CD-Si was about 7% due to the partial decomposition of β-CD immobilized on the surface of Cl-Si. The overall mass loss of Cl-Si in the range of 150–700 °C was 20.15%, which is mainly due to the decomposition of CPTES, so it can be concluded that the organic layer on Cl-Si was about 20.15%. The overall mass loss of 6-EA-β-CD-Si was 34.75%, mainly due to the thermal decomposition of β-CD and -CH_2_CH_2_CH_2_-, it is inferred that the amount of β-CD immobilized on the surface of Cl-Si is 34.75% (Neglecting -CH_2_CH_2_CH_2_- thermal decomposition). From the TG curve, it can be further concluded that 6-EA-β-CD-Si has a good thermal stability.

**Figure 4:**
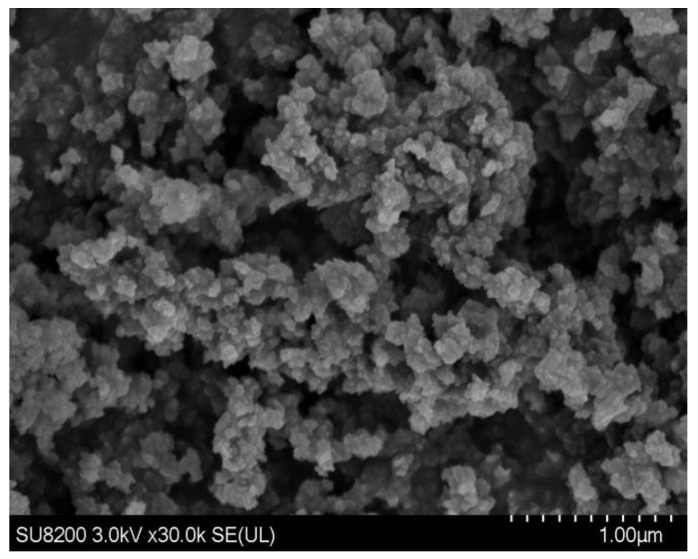
Scanning electron microscopy (SEM) images of 6-EA-β-CD-Si.

The scanning electron microscopy (SEM) image and particle size of 6-EA-β-CD-Si are employed to characterize the shape and dimensions of 6-EA-β-CD-Si (Figure 4). It can be clearly seen that 6-EA-β-CD-Si has a better spherical structure and a higher specific surface area, which increases the active sites of the guest molecules. In addition, the particle size of 6-EA-β-CD-Si is mainly distributed in the range of 150–250 nm, which is mainly due to the aggregation of nanoparticles. Exposed β-CD and active sites on the surface of 6-EA-β-CD-Si may be better contacted with MB, which is conducive to adsorption.

**Figure 5:**
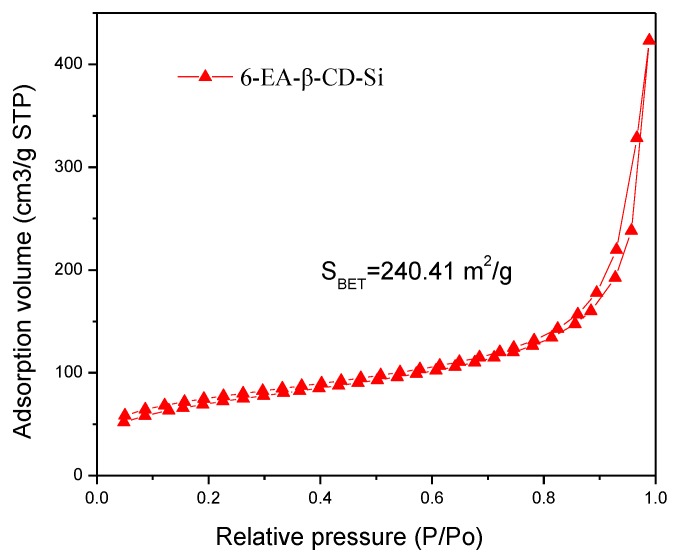
Brunauer-Emmett-Teller (BET) surface area of 6-EA-β-CD-Si.

From the results of Brunauer-Emmett-Teller (BET) surface area (Figure 5), it can be seen the adsorption curve and the desorption curve are almost in a coincide state, indicating that 6-EA-β-CD-Si has good adsorption properties. Meanwhile, β-CD, 6-EA-β-CD, Cl-Si, and 6-EA-β-CD-Si have been characterized in terms of BET surface area, hole volume, and pore size. Data are shown in Table 1. By comparison, 6-EA-β-CD has higher BET surface area and lower hole volume and pore size than β-CD, further verifying the successful modification of amine-ethyl on the β-CD surface. In addition, the pore diameter of 6-EA-β-CD-Si is larger than that of Cl-Si, further illustrating that 6-EA-CD-Si has both the pore size characteristics of cyclodextrin and the high BET surface area characteristics of Cl-Si. It’s noted that the BET surface area of 6-EA-β-CD-Si was 240.411 m^2^/g and average pore size is 4.162 nm, and the pore size distribution of the product is mostly between 2 and 10 nm, indicating that 6-EA-β-CD-Si belongs to a typical nanocomposite hole material. Compared with common adsorbent materials [30], the specific surface area of the two studies is the same, but this study has a higher adsorption capacity for MB. Compared with other cyclodextrin-modified materials [31], the specific surface area of the study can reach 919 m^2^/g, but its maximum adsorption capacity is only 60 mg/g. Thus, this novel material is conducive to adsorption, and the adsorption capacity of 6-EA-β-CD-Si is due to the existence of hydrophobic cavities and high specific surface area.

### 2.2. Adsorption Properties

**Figure 6:**
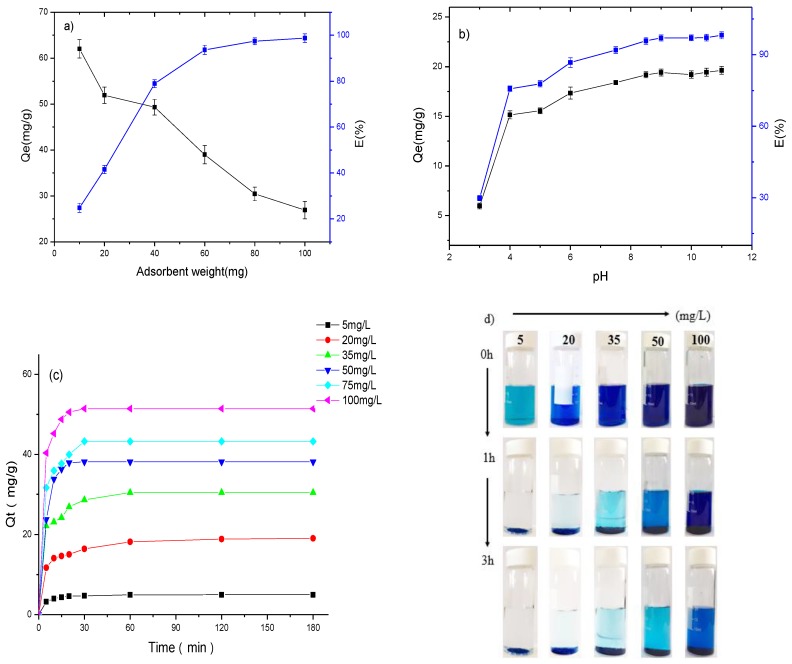
(**a**) Effective dosage of 6-EA-β-CD-Si on adsorption capacity of MB; (**b**) Effect of pH on adsorption of methylene blue (MB) by 6-EA-β-CD-Si; (**c**,**d**) MB solutions adsorbed by 6-EA-β-CD-Si at different incubation time.

For the adsorption reaction, effective dosage, pH, and initial MB concentration are the important factors affecting the adsorption of MB on 6-EA-β-CD-Si. Figure 6a shows the effects of 6-EA-β-CD-Si dosage on the adsorption of MB. With the increase of the dose of 6-EA-β-CD-Si, the removal rate of MB increases, but the dose of 6-EA-β-CD-Si increases further, and the removal rate increases slightly. And the amount of adsorption decreased with the increase of the dose, which is mainly due to the fact the higher dose provides a large excess of active sites, resulting in a lower utilization of the site at a certain concentration of MB [16]. Meanwhile, 6-EA-β-CD-Si aggregation and competition will also affect the adsorption behavior between 6-EA-β-CD-Si and MB.

pH value is also one of the important factors affecting the adsorption of dyes on the adsorbent material. It affects the surface charge of 6-EA-β-CD-Si and also affects the structure of MB in solution [32]. The pH of MB solution was varied from 3–12 to investigate the effect of pH value on the adsorption of 6-EA-β-CD-Si, and the results are shown in Figure 6b. The adsorption capacity of 6-EA-β-CD-Si onto MB was found to increase with a pH increase from 3 to 8. At about pH 8, the amount of adsorption displayed a slight increase. As pH changed from 8 to 12, the maximum removals in the range of 98.5 to 99.2% were achieved in the range of 9 to 12, indicating that the alkaline conditions conducive to the adsorption. This is due to the excess hydrogen ions (H^+^) competed with MB for the active sites on the surface of 6-EA-β-CD-Si [19], hindering the adsorption of 6-EA-β-CD-Si onto MB. As the pH increases, the dimethylamine group becomes neutral, enhancing the interaction of MB molecules with the surface of 6-EA-β-CD-Si.

Figure 6c–d shows the amount of MB adsorbed (Qt, mg/g) at a specific reaction time (t, min) at pH 8 (± 0.1) by 6-EA-β-CD-Si at temperature 30 °C, respectively. It can be seen that the adsorption amounts of 6-EA-β-CD-Si onto MB increased with increasing incubation time. The adsorption amount increases rapidly within 0–10 min, the adsorption amount increases slowly within 10–60 min, and the adsorption equilibrium was reached at an incubation time of 60 min. Therefore, in about 10 min, the adsorption rate of 6-EA-β-CD-Si onto MB can reach about 80% at equilibrium, so the fast adsorption rate indicates that the internal mass transfer resistance is weak during the adsorption process [30]. The short adsorption equilibrium time indicates the high removal efficiency of 6-EA-β-CD-Si, which is significant in practical applications. In addition, with the initial concentration of MB increasing from 20 mg/L to 100 mg/L, the adsorption capacity increases from 18.86 to 51.42 mg/g, indicating that the higher initial concentration will provide a more powerful driving force to overcome the mass transfer resistances between 6-EA-β-CD-Si and the MB solution.

### 2.3. Adsorption Kinetics

In order to evaluate the rate-controlling step in the adsorption of MB, the data were fitted by Pseudo-first-order kinetic model (1) and Pseudo-second-order kinetic model (2), respectively.

Pseudo-first-order kinetic model:

(1) $$\ln\left(\mathrm{Qe}-\mathrm{Qt}\right)=\mathrm{lnQe}-K_{1}t$$

where Qe and Qt are the adsorption capacities at equilibrium time and time t (mg/g), respectively. K_1_ represents the pseudo-first-order kinetic model rate constant (1/min). K_1_ and Qe can be gained from the slope and intercept of the ln(Qe − Qt) versus t, respectively.

Pseudo-second-order kinetic model:

(2) $$\;t/Q_{t}=-\frac{1}{K_{2}{\mathrm{Qe}}^{2}}+\frac{1}{\mathrm{Qe}}t$$

where K_2_ represents the pseudo-second-order kinetic model rate constant (g/(mg min)). Qe and K_2_ can be obtained from the slope and intercept of the t/Qt versus t, respectively.

**Figure 7:**
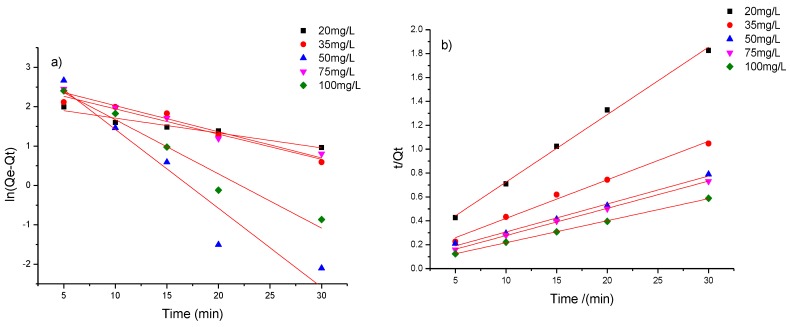
The linear fitting of Pseudo-first-order (**a**) and Pseudo-second-order (**b**) for removal of MB.

The adsorption kinetics of 6-EA-β-CD-Si were conducted with the initial concentration of MB from 20 mg/L to 100 mg/L and initial pH of 8 (± 0.1). The linear fitting results for the adsorption of MB on 6-EA-β-CD-Si are shown in Figure 7a,b. Based on the values of the linear regression coefficient (R^2^), the adsorption process of MB by 6-EA-β-CD-Si was fitted better by the pseudo-second-order kinetic model (R^2^ = 0.9896 − 0.9994) than by the pseudo-first-order kinetic model (R^2^ = 0.9024 − 0.9597). Furthermore, the kinetic parameters, which were calculated, are shown in Table 2. The adsorption capacity of the experimental values (Qe, exp) is very close to the value calculated from the pseudo-second-order kinetic model (Qe, cal) at five different initial concentrations. The rate constant (K_2_) of pseudo-second-order kinetic model decreases with the initial concentration increasing from 20 mg/L to 100 mg/L. Therefore, the reaction rate is inversely proportional to the initial concentration of MB.

### 2.4. Adsorption Isotherms

Adsorption isotherms can also better simulate the adsorption behavior of MB. The Langmuir adsorption isotherm model (Equation (3)) and the Freundlich adsorption isotherm model (Equation (4)) have been used to discuss the equilibrium characteristics of the adsorption process. 

The Langmuir isotherm equation can be written as

(3) $$\;\frac{\mathrm{Ce}}{\mathrm{Qe}}\;=\;\mathrm{Qmax}\;\times \;\mathrm{Ce}+\frac{1}{\left({\mathrm{Qmax}\;\times \;K}_{L}\right)}$$

where Ce is the equilibrium concentration of the MB solution (mg/L), Qe is the adsorption capacity of MB on the adsorbent at equilibrium (mg/g), Qmax is the maximum adsorption capacity (mg/g), and K_L_ is the Langmuir binding constant (L/mg), which is related to the free energy of adsorption. The value of Qmax and K_L_ can be calculated from the slope and intercept of the Ce/Qe versus Ce, respectively.

Freundlich isotherm equation can be written as

(4) $$\mathrm{lgQe}\;=\;\frac{1}{n}{\mathrm{lgCe}\;+\;\mathrm{logK}}_{F}$$

where K_F_ is the Freundlich parameter and n is constant related to adsorption intensity. If n is greater than 1, the adsorption process is favorable. K_F_ and n can be derived from the slope and intercept of the lgQe versus lgCe.

**Figure 8:**
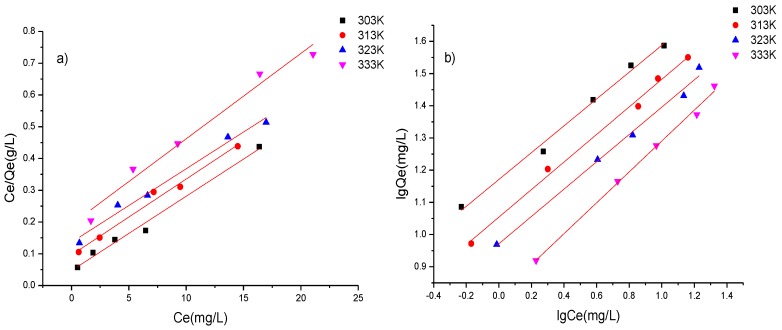
The equilibrium isotherms for MB adsorbed by 6-EA-β-CD-Si: (**a**) the Langmuir model; (**b**) the Freundlich model.

The equilibrium adsorption study was performed with different initial concentrations of MB at the temperatures 303 K, 313 K, 323 K, and 333 K, respectively. The slope and the intercept of each linear plot shown in Figure 8a, b. It can be found that the regression coefficient (R^2^) obtained from Freundlich model (R^2^ = 0.9903 − 0.9944) is much higher than that from Langmuir model (R^2^ = 0.9707 − 0.9858) for MB. The results suggested that the Freundlich isotherm fits better with the experimental data. The adsorption process between MB and material could occur on heterogeneous surfaces without being restricted to monolayers, and as the concentration of MB increase, more MB molecules can be captured on the material surfaces. Furthermore, the results fitted by Freundlich and Langmuir models are shown in Table 3. As the temperature increases, the maximum adsorption capacity decreases. The Qmax calculated from the Langmuir model is 39.37, 39.21, 36.90, and 36.36 mg/g at the temperatures 303, 313, 323, and 333 K, respectively. Therefore, it is concluded that the temperature remarkably influences the adsorption of MB, which indicates that adsorption is an exothermic process. Meanwhile, n is greater than 1 (n = 2.0669 − 2.8775), intimating that the adsorption of MB on 6-EA-β-CD-Si is a favorable process [33].

### 2.5. Adsorption Thermodynamics

**Figure 9:**
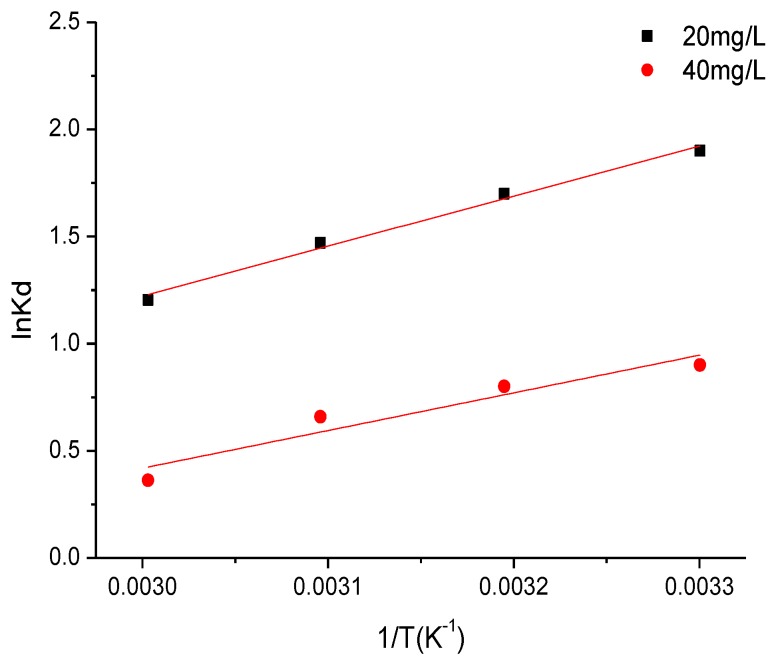
Liner plot of lnK_d_ versus 1/T for the adsorption of MB.

Temperature is also a controlling parameter for MB removal. To determine the effect of 6-EA-β-CD-Si on the adsorption of MB at different temperatures, thermodynamic parameters need to be further determined and calculated by the following formula (Equations (5)–(7)). The values of enthalpy change (ΔH°) and entropy change (ΔS°) were obtained from the slope and intercept, respectively, of the graphical plot of lnKd as a function of 1/T (Figure 9).

(5) ΔG°=−RTlnKd

(6) $$\mathrm{lnKd}=-\left(\frac{\Delta H{}^{\circ}}{R}\right)\left(\frac{1}{T}\right)+\frac{\Delta S{}^{\circ}}{R}$$

(7) ΔG°=ΔH°−TΔS°

where ΔG° is the Gibbs free energy variable for the adsorption process, kJ/mol; Kd is the isothermal adsorption constant (L/g); Kd = Qe/Ce; R is the universal gas constant, 8.314 J/(mol·K); T is the thermodynamic temperature, K; ΔH° is the enthalpy change of the adsorption process, and kJ/mol; ΔS° is the entropy change of the adsorption process, J/(mol·K).

Table 4 presents the thermodynamic parameters at the initial concentration of 20 mg/L and 40 mg/L in MB aqueous solution. Values of the enthalpy changes (ΔH° < 0 kJ/mol), indicating that the adsorption of MB is an exothermic process, which is a good explanation for the decrease in the amount of adsorption with rising temperature appears in Table 3, and their magnitudes (<40 kJ/mol) means the adsorption process is mainly due to the physical adsorption in nature and the weak contact in the system [34]. The Gibbs free energy changes (ΔG°) are negative of the tested temperatures range from 303 K to 333 K, confirming that the adsorption of MB on 6-EA-β-CD-Si is spontaneous and thermodynamically favorable [35]. The values of ΔG° are among −15.6178 and −11.2937 kJ/mol, which further indicating that the physical adsorption might dominate the adsorption of MB on 6-EA-β-CD-Si, along with the weak interactions between MB and 6-EA-β-CD-Si.

### 2.6. Adsorption Mechanism

**Figure 10:**
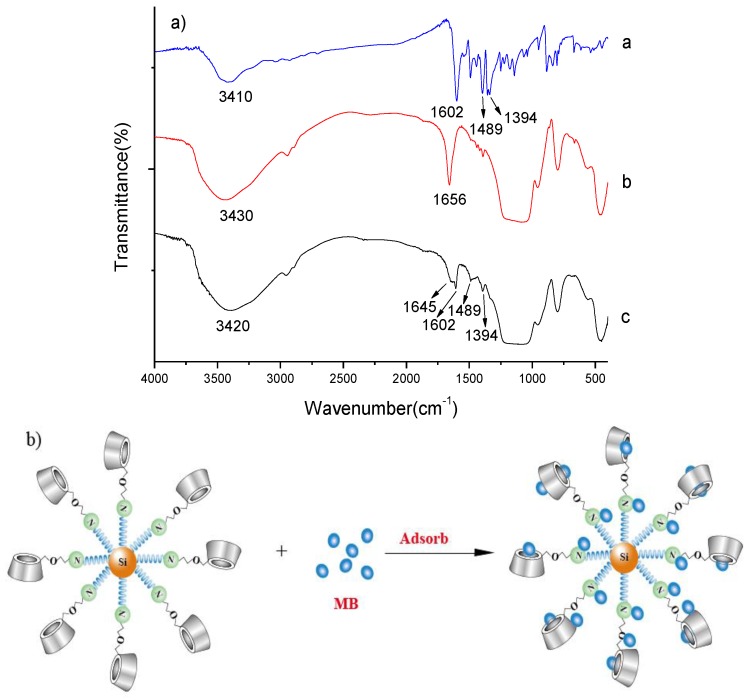
(**a**) FT-IR spectroscopy of (a) MB, (b) 6-EA-β-CD-Si, (c) 6-EA-β-CD-Si/MB; (**b**) Speculated adsorption mechanism of MB onto 6-EA-β-CD-Si.

Through the above study of adsorption kinetics, adsorption isotherms, thermodynamic analysis, and optimum pH conditions, the MB adsorption mechanism can be inferred. To verify the adsorption mechanism, the FT-IR spectra of MB, 6-EA-β-CD-Si, and 6-EA-β-CD-Si/MB are shown in Figure 10a. After the adsorption of MB onto 6-EA-β-CD-Si, the FT-IR spectra exhibits many changes. It is found that the appearance of peaks at 1602 cm^−1^, 1489 cm^−1^, and 1394 cm^−1^ in the FT-IR spectra of 6-EA-β-CD-Si after adsorption of MB, which belongs to the stretching vibrations of C=C in benzene rings and the C-N stretching vibration existing on the MB molecules. Thus, there is the interaction between MB and 6-EA-β-CD-Si, which is a hydrogen bond. The adsorption mechanism of MB onto 6-EA-β-CD-Si can be summarized as shown in Figure 10b. Based on the analysis above, the adsorption mechanism is attributed to the host-guest interactions between cyclodextrin and aromatic molecules, the electrostatic interactions, and weak hydrogen bonding between MB and 6-EA-β-CD-Si.

Table 5 contrasts this study on the adsorption of MB to those in previous literature and revealed that 6-EA-β-CD-Si shows a greater adsorption capability comparing with other adsorbents, including activated carbon and some other low-cost adsorbents. However, the adsorption mechanisms between MB and the adsorbents reported by other researchers were unknown. In this work, through the detailed discussion, it can be found that the removal mechanism of MB by 6-EA-β-CD-Si is mainly due to the host–guest interactions, the electrostatic interactions, and weak hydrogen bonding between 6-EA-β-CD-Si and MB. These results indicated that the 6-EA-β-CD-Si core-shell nanomaterial could be considered as a promising adsorbent for the removal of MB from wastewater. At the end of the third cycle, the adsorption rate of MB on 6-EA-β-CD-Si can still reach 65% (data not shown). Furthermore, the processes of adsorption, separation and desorption with 6-EA-β-CD-Si avoids the secondary pollution of water and improve the adsorption efficiency of dyes compared with the previous.

## 3. Materials and Methods

### 3.1. Preparation of 6-EA-β-CD

2-chloroethylamine hydrochloride (1.5 g) was sonicated and dispersed in double-distilled water (6 mL). β-CD (15 g) was added to the dispersion and dried at 50 °C until the water was completely evaporated. The product was added to NaOH solution (10%), and the reaction was stirred for 240 min at 70 °C. After the reaction, the product was precipitated with methanol, filtered and washed three times and dried at 40 °C for 6 h to obtain 6-EA-β-CD. 

### 3.2. Preparation of Cl-Si

The synthesis of Cl-Si was slightly modified according to the method previously reported [26]. 3-Chloropropyl-triethoxysilane (CPTES) (4.78 mL) and tetraethyl orthosilicate (TEOS) (22 mL) were fully dissolved in anhydrous ethanol (60 mL). Ultrapure water (7.2 mL) and 1 M tetrabutylammonium fluoride in THF (6 mL) were dissolved in another absolute ethanol solution (40 mL), and then the two mixtures were thoroughly mixed for 10 s to obtain a homogenous solution. Gel was observed after 30 min and aged at room temperature for six days. The obtained gel was crushed, filtered, and washed successively with ethanol and acetone solution. Thus, the material was dried at 80 °C for 6 h to obtain Cl-Si as a white powder.

### 3.3. Preparation of 6-EA-β-CD-Si

The 6-EA-β-CD-Si was prepared as following methods: 6-EA-β-CD (3 g), Cl-Si (2 g), and KI (0.2 g) were dissolved in DMF (60 mL), nitrogen gas was blown into the reaction vessel for 5 min so that air in the reaction vessel was discharged, and the reaction was stirred at 110 °C for 24 h. After the reaction was completed, the mixtures were cooled to room temperature, and the suspension was filtered and washed successively with DMF, ethanol, and water, and then the obtained material was dried under vacuum at 60 °C for 9 h to obtain 6-EA-β-CD-Si as a white power.

### 3.4. Samples Characterization

Fourier transform infrared spectrometer was carried out using a Nicolet 5700 spectrometer (Thermo Fisher Scientific, Waltham, MA, USA), and spectra were recorded between 500 and 4000 cm^−1^. The X-ray diffraction spectrometer (XRD) experiments were recorded on an X-ray diffraction spectrometer using Cu Kα 1.5406 Å in the scattering angle range of 2*θ* = 5 to 50°. The SEM images of 6-EA-β-CD-Si were taken using a scanning electron microscope. Thermal stability was analyzed by a thermogravimetric analyzer in the air atmosphere with the airflow rate of 50 cm^3^/min. The temperature was ranged from 30 to 700 °C at a scanning rate of 10 °C/min. The Brunauer-Emmett-Teller (BET) surface area analyses of the sample were performed using a low-temperature N_2_ adsorption-desorption technique with a Micromeritics ASAP 2020 (Micromeritics, Orlando, FL, USA) gas adsorption apparatus. The particle size of as-synthesized nanoparticles was measured using a Nanoparticle size and zeta potential analysis Uptake was determined by measuring the reduction in absorbance or depletion from solution using UV-vis spectrophotometry at the wavelength 660 nm.

### 3.5. Adsorption of Anionic Dyes

The adsorption behaviors of 6-EA-β-CD-Si were systematically investigated by changing the factors of adsorbent dosage, pH value, adsorption time, initial concentration, and adsorption temperature. The specific experimental process is as follow: The process of adsorption was performed in bath thermostatic shaker with a certain temperature at a speed of 210 rpm. After the adsorption reaction was completed, the supernatant solution was taken after centrifugation at 10,000 rpm for 15 min, and the residual concentration of MB was determined by UV-vis spectrophotometry at the wavelength 660 nm.

The adsorption capacity Equation (8) and removal rate Equation (9) were calculated according to the following formulas:

Qe = (C_0_-C_e_)/C_0_ × V/m (8)

E(%) = (C_0_-C_e_)/C_0_ × 100 (9)

where Qe (mg/g) is the adsorbed amount of MB on 6-EA-β-CD-Si. C_0_ (mg/L) and Ce (mg/L) are the initial and real-time concentrations of MB, respectively. V (mL) is the volume of solution, and m (mg) is the mass of 6-EA-β-CD-Si used.

## 4. Conclusions

In summary, a novel cyclodextrin-functionalized hybrid silicon nano-adsorbent core-shell material (6-EA-β-CD-Si) was successfully prepared via the nucleophilic substitution method, and the removal of MB in an aqueous solution was studied. The adsorption results confirm that 6-EA-β-CD-Si is an effective adsorbent and exhibited good adsorption property toward methylene blue (MB). The best pH value for adsorption of MB is 10.5 with maximum removal efficiency of 99.2%. The result of kinetic study shows that pseudo-second-order kinetic model is the best kinetic model for describing of MB adsorption onto 6-EA-β-CD-Si and maximum 99.5% removal was achieved during adsorption kinetic study. The equilibrium data were well-modeled by the Freundlich isotherm model, and the thermodynamic parameters indicated that the adsorption process of MB onto 6-EA-β-CD-Si was a spontaneous and exothermic process. The host-guest interactions between 6-EA-β-CD-Si and aromatic molecules, the electrostatic interactions, and weak hydrogen bonding between MB and 6-EA-β-CD-Si are essential for the adsorption of MB. The above results confirmed the potential of 6-EA-β-CD-Si as an efficient adsorbent material with advantages of widespread availability, environmental friendliness, and low cost in separating dye from solution. Thus, 6-EA-β-CD-Si has great potential applications in industrial wastewater treatment and environmental protection.

## Figures and Tables

**Table 1:** Micro-structure data of β-CD, 6-EA-β-CD, Cl-Si, and 6-EA-β-CD-Si.

Material	BET Surface Area (m^2^/g)	Hole Volume (cm^3^/g)	Pore Size (nm)
β-CD	0.3445	0.0003	12.00
6-EA-β-CD	0.3669	0.0002	10.80
Cl-Si	341.7485	0.2438	3.940
6-EA-β-CD-Si	240.4112	0.3010	4.162

**Table 2:** Kinetic parameters of different initial concentrations of MB.

C_0_ (mg/L)	Pseudo-first-order Kinetic Model			Pseudo-second-order Kinetic Model			
	K_1_/min^−1^	Qe, cal/(mg/g)	R^2^	K_2_/min^−1^	Qe, cal/(mg/g)	R^2^	Qe, exp/(mg/g)
20	0.0378	8.0776	0.9391	0.02	17.7305	0.9966	18.8629
35	0.064	13.2567	0.9387	0.0106	31.0559	0.9896	30.4405
50	0.2004	30.8674	0.9024	0.0075	42.7351	0.9931	39.122
75	0.0664	14.7508	0.9597	0.0051	43.8596	0.9993	43.233
100	0.1377	21.0774	0.9549	0.0034	54.3478	0.9994	51.4216

Qe, cal is the calculated equilibrium adsorption capacity, and Qe, exp is the experimental equilibrium adsorption capacity.

**Table 3:** Langmuir and Freundlich isotherm fitting parameters at different temperatures.

T(K)	Langmuir Adsorption Isotherm			Freundlich Adsorption Isotherm		
	Qmax (mg/g)	K_L_ (L/g)	R^2^	K_F_	n	R^2^
303	39.47	0.4790	0.9858	15.8433	2.8775	0.9927
313	39.21	0.3778	0.9825	12.4202	2.6749	0.9930
323	36.90	0.2691	0.9812	10.4340	2.6142	0.9903
333	36.36	0.1436	0.9707	6.5337	2.0669	0.9944

**Table 4:** Adsorption thermodynamic parameters at different temperatures.

Co (mg/L)	T (K)	ΔG° (kJ/mol)	ΔH° (kJ/mol)	ΔS° (J·mol^−1^·K^−1^)
20	303	−15.6178	−19.3966	−12.4710
	313	−15.4931		
	323	−15.3684		
	333	−15.2437		
40	303	−11.5930	−14.616	−9.9768
	313	−11.4933		
	323	−11.3935		
	333	−11.2937		

**Table 5:** Adsorption isotherms of MB on various adsorbents.

Adsorbents	BET Surface Area (m^2^/g)	Isotherm	Adsorption Capacity, Qm (mg/g)	pH	Reference
sodium hydroxide	31.35	Langmuir	27.86	–	[36]
phenyl-functionalized silica materials	−	Sips	33.7	8	[37]
magnetic graphene-carbon nanotube	–	Langmuir	24.88	7	[38]
clay	30	Freundlich	6.3	–	[39]
activated carbon from waste biomass	240.02	Langmuir	16.43	6	[30]
vegetal fiber activated carbons	–	–	33.7	10	[40]
cyclodextrin-functionalized hybrid silicon	240	Freundlich	39.47	8	This work
